# Toward unbiased assessment of treatment and prevention: modeling household transmission of pandemic influenza

**DOI:** 10.1186/1741-7015-10-118

**Published:** 2012-10-09

**Authors:** Gerardo Chowell, Hiroshi Nishiura

**Affiliations:** 1 School of Human Evolution and Social Change, Arizona State University, Tempe, AZ, USA; 2 Division of Epidemiology and Population Studies, Fogarty International Center, National Institutes of Health, Bethesda, MD, USA; 3 School of Public Health, Li Ka Shing Faculty of Medicine, The University of Hong Kong, Hong Kong Special Administrative Region, China; 4 PRESTO, Japan Science and Technology Agency, Saitama, Japan

**Keywords:** epidemic, estimation, household transmissibility, household transmission studies, mathematical model, outbreaks, pandemic, reproduction number, secondary attack rate, serial interval

## Abstract

Providing valid and reliable estimates of the transmissibility and severity of pandemic influenza in real time is key to guide public health policymaking. In particular, early estimates of the transmissibility are indispensable for determining the type and intensity of interventions. A recent study by House and colleagues in *BMC Medicine *devised a stochastic transmission model to estimate the unbiased risk of transmission within households, applying the method to datasets of the 2009 A/H1N1 influenza pandemic. Here, we discuss future challenges in household transmission studies and underscore the need to systematically collect epidemiological data to decipher the household transmission dynamics. We emphasize the need to consider three critical issues for future improvements: (i) capturing age-dependent heterogeneity within households calls for intensive modeling efforts, (ii) the timeline of observation during the course of an epidemic and the length of follow-up should be aligned with study objectives, and (iii) the use of laboratory methods, especially molecular techniques, is encouraged to distinguish household transmissions from those arising in the community.

See related article: http://www.biomedcentral.com/1741-7015/10/117

## Background

Valid and reliable estimates of the transmissibility and severity of an unfolding influenza pandemic are key to guide public health intervention efforts, such as timely antiviral treatment of symptomatic individuals and social distancing measures [1]. Applying mathematical modeling methods to empirically-observed epidemiological datasets has played an essential role in providing the world with statistical estimates of these key epidemiological quantities. In particular, transmissibility estimates of pandemic influenza in real time are indispensable for determining the type and intensity of interventions and are used as an indicator for public health policymaking during both containment and mitigation phases [2]. The transmissibility of influenza at a community setting has been commonly measured by employing the reproduction number, *R*, defined as the expected number of secondary cases generated by a typical primary infectious individual in a population that may be partially susceptible due to prior exposure to similar viruses or vaccination campaigns [3].

Because about one-third of all influenza secondary transmission events are believed to occur within households [2], estimating the risk of transmission in the household setting is crucial for interpreting household epidemiological data and guiding household-based interventions [4,5]. Household transmission studies offer an opportunity to quantify the conditional risk of infection given an exposure and allow us to observe a wide spectrum of disease without ascertainment bias. Households provide an ideal transmission unit to quantify any relative differences in susceptibility and infectiousness, thereby allowing the quantification of vaccine efficacy and effectiveness of various interventions. In addition, the household serial interval, that is, the average time between illness onsets of successive cases in a transmission chain among household members, is another key epidemiological quantity that has been estimated from household transmission studies and used to translate the epidemic growth rate into the reproduction number. A number of household transmission studies were conducted during the 2009 A/H1N1 influenza pandemic with the goal of characterizing the transmission dynamics [5]. Most studies so far have used influenza-like illness (ILI) and/or laboratory confirmed cases to make inferences on household secondary attack risks, the former being not specific for influenza and the latter missing a substantial amount of infected individuals. To offer additional insights into the transmission dynamics of 2009 A/H1N1 influenza, House and colleagues in *BMC Medicine *[6] devised a stochastic epidemic model that explicitly accounts for differential case definitions to estimate the risk of transmission within households. Here, we aim to identify pros and cons of the proposed novel approach and suggest new ways to move forward household studies.

## A novel framework for estimating severity and transmissibility of 2009 A/H1N1

Given limited number of useful methods to analyze household transmission data of influenza, House and colleagues went one important step forward. Specifically, they provided a framework that connects the final state of a stochastic epidemic model with a statistical estimation approach so that one can infer the risk of transmission within households using the data stratified by household size, while accounting for differential levels of case ascertainment. Case ascertainment is particularly important when not all suspected cases are laboratory tested for influenza or other respiratory viruses. In the House *et al*. study [6], the risk of household transmission, denoted by *T*, is theoretically regarded as a less biased measure of household transmissibility than the observed 'crude' secondary attack risk (that is, the proportion of household secondary cases among the total of susceptible household members). This is because the final size model using *T *addresses multiple chains of transmission in households and the dependence of the risk of infection between households [7]. Using the parameter *T*, one may be able to assess the transmissibility in households without serious bias, such as, for example, those arising from household structure (for example, size and membership), community risk, and tertiary transmission or additional chains of transmission in households. To illustrate their estimation framework, House *et al*. [6] used an epidemiological dataset comprising 424 index cases from 424 separate households and their 1612 household contacts in Birmingham, one of the first cities in the UK to be affected by the 2009 pandemic. An overall secondary attack risk of infection was calculated at 39.7% (95% CI 34.9 to 44.0). They also showed that transmission risk at the household level based on laboratory confirmed A/H1N1 cases would be underestimated. A negative correlation between the transmission probability and household size was also identified. The authors also conducted a review of household transmission studies of 2009 A/H1N1 influenza, identifying large variation in estimates of *T *and secondary attack risks, which could be attributed to differences in household size distribution, underlying demographic characteristics (such as age structure), case ascertainment, and the effects of changes in population behaviors and specific public health interventions [5].

To the best of our knowledge, the study by House *et al*. [6] is the first to use statistical methods to integrate the final size equation, derived by Ball [7], with empirical household transmission data stratified by household size. Compared to classical models such as those based on chain binomial model or those separating household transmission risk from community risk of infection [4], the series of studies by Ball and his colleagues clearly addressed the dependence of the risk of infection between households, showing that the so-called community risk of infection is explained by the household size distribution in a community and distribution of infected individuals in those households. In their statistical estimation approach, House and colleagues jointly estimated the transmission probability and the diagnostic performance parameters of differential case definitions to better integrate all the epidemiological data available. Achieving such joint estimation will eventually permit us to precisely estimate the efficacy of antiviral treatments and vaccination without suffering from ascertainment bias.

## Future directions and conclusions

What are the unresolved and future challenges? First of all, capturing household level transmission dynamics requires further elaboration of a key epidemiological aspect. While the study by House *et al*. [6] accounted for variability in household size and differences in case outcomes from epidemiological data, describing the transmission dynamics of pandemic influenza requires us to look into the age-dependent heterogeneity. In particular, the well-known role of school age children in rapidly disseminating influenza (including those within households [8]) calls for age-specific transmission parameters. Although it is possible to capture the age-specific dynamics using a simple household model [9], explicitly incorporating school transmission into an explicit Ball-type model is likely to require a model structure with three levels of mixing. Second, epidemiological study designs need to be reconsidered to collect useful data to satisfy specific study objectives. During the 2009 pandemic, a number of household studies only gathered household transmission data for a limited period of time, especially during the early stages of the pandemic. In this case, observed data may not represent the final epidemic state, which could lead to bias when estimating the household transmissibility. In other words, the final size equation connected to the corresponding stochastic model makes an unsupported assumption on having captured the unobserved full transmission process. Moreover, the time period to follow-up households is often restricted to the first 7 days following symptoms onset in the index case [10,11]. Hence, the observation timeline during the course of a pandemic and the duration of follow-up should be aligned with study objectives. The observation setting also complicates the interpretation of household transmission data, most notably those collected during active surveillance (for example, through containment efforts). Third, we suggest that future household transmission studies employ laboratory methods (for example, genotyping) to help researchers disentangle within-households transmission events and explicitly track the network of transmission links. An effort in this direction in the context of the 2009 pandemic was carried out to capture only secondary cases arising within households [12]. Moreover, statistical methods could be employed to characterize the latent period and asymptomatic ratio from transmission links inferred from the observed transmission network.

## Conclusions

In summary, novel mathematical modeling tools based on carefully designed epidemiological studies for data collection in confined settings have the potential to deepen our understanding of the ecoepidemiology of influenza and other emerging and re-emerging infectious diseases. Advancing inferential techniques can help estimate the individual effect of treatment and prevention without ascertainment bias for mild disease. Thus, it is essential to critically review the practical objectives of household studies, the corresponding study designs and the corresponding modeling assumptions in a systematic manner.

## Competing interests

The authors declare that they no competing interests

## Authors' contributions

Both authors contributed to the writing and editing of this commentary and have read and approved the final manuscript.

## Authors' information

GC is an associate professor in the School of Human Evolution and Social Change at Arizona State University and a research fellow at the Fogarty International Center, US National Institutes of Health. His research interests include mathematical and statistical modeling of infectious disease transmission and control interventions, with a focus on seasonal and pandemic influenza and the quantitative characterization of past influenza pandemics. HN is an assistant professor of the School of Public Health, The University of Hong Kong. He contributes to the theoretical foundations of infectious disease epidemiology by employing various types of mathematical models including multistate, multihost, multistrain and multilayer structured models with particular emphasis on their implications to statistical analysis of infectious disease data.

## Pre-publication history

The pre-publication history for this paper can be accessed here:

http://www.biomedcentral.com/1741-7015/10/118/prepub
